# Next-generation sequencing-assisted diagnosis of a case of leprosy misdiagnosed as erythema multiforme

**DOI:** 10.1186/s12941-022-00532-4

**Published:** 2022-09-07

**Authors:** Yanfang Zhang, Xuezhong Lei, Jiajie Lu

**Affiliations:** Center of Infectious Diseases, West China Hospital, Sichuan University, Chengdu, 610041 Sichuan Province China

**Keywords:** Leprosy, Treatment, Special pathological staining, Second-generation gene sequencing, Case report

## Abstract

**Background:**

Leprosy is a chronic infectious disease caused by *Mycobacterium leprae* or *Mycobacterium lepromatosis* that is mainly transmitted through droplets from the nose and mouth of untreated patients. Owing to the lack of specific serological markers and clinical manifestations, leprosy can be easily confused with other skin lesion-related diseases and is difficult to distinguish.

**Case presentation:**

This study introduces and summarises the diagnosis and treatment process of a case of leprosy misdiagnosed as erythema multiforme for a long time. A 43-year-old female was admitted to our hospital because of “repeated fever with superficial lymphadenopathy and systemic rash in May”. The diagnosis of the patient was based on the two main clinical characteristics of superficial lymphadenopathy and systemic pleomorphic erythema by using a combination of multiple samples of lymph nodes and skin, routine pathological examination, immunohistochemistry, acid-fast, silver hexamine, periodic acid-Schiff (PAS) staining, and second-generation gene sequencing of fresh biopsy tissue. The patient was treated with dapsone, rifampicin, and clofazimine at the Institute of Dermatology and Venereal Diseases. After treatment for 1 year, her temperature returned to normal, the area of facial erythema decreased, and the volume of axillary lymph nodes had gradually reduced.

**Conclusions:**

In conclusion, special pathological staining and second-generation gene sequencing show promising advantages in distinguishing leprosy from other skin lesion-related diseases.

## Background

Leprosy is a chronic infectious disease caused by *Mycobacterium leprae* or *Mycobacterium lepromatosis* [1] and is mainly transmitted by droplets from the nose and mouth of untreated patients [2].These pathogens are usually detected in the sub-epidermal zone, inside the sweat glands, arrector pili muscle, Schwann cells, macrophages, and around hair follicles [3], resulting in skin injury and loss of sensorimotor function [4]. The incubation period of leprosy is long, approximately 4–5 years, and some symptoms may not appear for 10 or even 20 years. Nevertheless, some cases have been reported in babies [5] and toddlers [6]. Clinically, three cardinal signs are recommended to diagnose leprosy, including loss of sensation in a skin lesion, enlarged peripheral nerve, and positive skin smears [7], and leprosy is normally diagnosed when a patient shows two of these three cardinal signs. In endemic countries, the WHO considers one cardinal sign sufficient [7]. Moreover, the clinical manifestations of leprosy are extensive and nonspecific because of the complex immune response to the pathogen; lesions can involve the skin, mucosa, and peripheral nerves. Some patients exhibit nerve involvement or a skin lesion that appears irregularly or disappears naturally, whereas others show a variety of skin lesions accompanied by multiple nerve stem damage and even involvement of internal organs such as the eyes, pharynx, liver and bone [7]. Age and sex are important risk factors for leprosy: adolescents aged between 10 and 19 years and adults aged more than 30 years are more exposed to leprosy, and the possibility of infection in adult males is twice as high as that in adult females [8].

At present, leprosy is prevalent in India, Indonesia, and Brazil, accounting for most newly discovered cases. The distribution of leprosy in China is uneven; southwestern areas such as Yunnan, Guizhou, and Sichuan Province are the main incidence areas. At present, the prevalence of leprosy in China is approximately 0.235/100,000 [4, 4]. Since the World Health Organization (WHO) recommended chemotherapy (MDT) for the treatment of leprosy in 1982, treatment of leprosy has achieved satisfactory results, and the global prevalence has decreased significantly. In 2013, the prevalence rate of leprosy was reduced to 0.32/10,000 worldwide. In the same year, the WHO removed China from the list of countries with a high prevalence of leprosy [9]. Therefore, leprosy has gradually become a rare disease in China. Because leprosy has no specific serological markers or clinical manifestations, it is easily confused with other skin lesion-related diseases and is difficult to identify. In areas with underdeveloped medical facilities, delayed diagnosis and incorrect treatments can cause substantial medical expenses and waste of medical resources. Consequently, delayed treatment of leprosy-related nerve damage may lead to permanent limb deformities, dysfunction, or even death [10]. This paper introduces and summarises the diagnosis and treatment process of leprosy misdiagnosed as erythema multiforme for a long time and provides new insight for the clinical diagnosis of leprosy.

## Case presentation

**Fig. 1:**
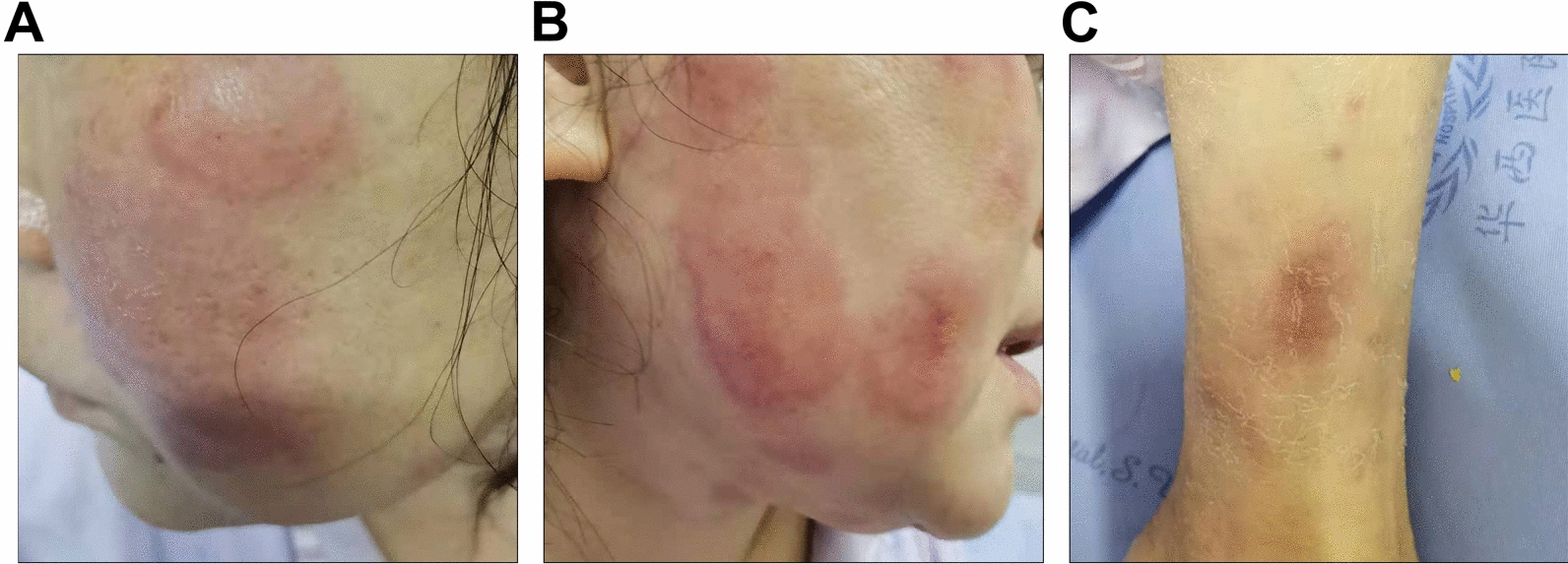
Skin lesions on the patient’s face (**A** and **B**) and right lower leg (**C**)

A 43-year-old female professional technician from Nanbu County, Sichuan Province, was admitted to our hospital on 1 September 2020 with complaints of “repeated fever with superficial lymphadenopathy and systemic rash for 5 months.” The patient had no obvious cause of fever for 5 months before admission, with a maximum temperature of 41 °C. Multiple subcutaneous nodules with a diameter of approximately 1 cm could be palpated in the neck, armpit, and groin and were accompanied by multiple rashes on the face and body (Fig. 1). There was no pain, itching, or burning sensation while walking; no hypoesthesia of the eyes, hands, or feet; no movement disorder of the limbs; no chills or shivering; no headache or dizziness; no chest tightness or shortness of breath; and no abdominal distension or pain. After treatment with ibuprofen in the local clinic, her body temperature temporarily normalised but repeatedly rose again. A left inguinal lymph node biopsy was performed at a local hospital. Histopathological examination revealed lymphoid tissue hyperplasia, granulomatous structures with necrosis, and immunohistochemical staining for histiocytic necrotising lymphadenitis. The patient was diagnosed with histiocytic necrotising lymphadenitis and pleomorphic erythema. Therapy including levofloxacin and prednisone acetate (30 mg/day) was administered. Subsequently, the patient's temperature and systemic erythema decreased; thus, levofloxacin was stopped, prednisone acetate was reduced to 20 mg/day, treatment was maintained, and the patient was discharged from the hospital. At 1 month before admission, the patient again had a fever, and her temperature did not significantly decrease after treatment in the local clinic. As a result, the patient visited our hospital. Physical examination on admission indicated the following: body temperature 36.9 °C, pulse 93 beats/min, respiration 20 breaths/min, and blood pressure 106/78 mmHg. Visible and palpable enlarged lymph nodes under the chin, bilateral armpits, and groin were approximately 1 cm in diameter, hard, tender, and fixed. Pleomorphic erythema was present on the face and over the body. The lesions were approximately 2–7 cm in size, with some protruding from the skin but not accompanied by ulceration or secretion. Physical examination of the heart, lung, abdomen, perineum, spine, limbs, and nervous system revealed no abnormalities.

Blood examination after admission showed that the total number of leukocytes was 7.94 × 10^9^/L, the percentage of neutrophils was 85.6%, and the absolute value of neutrophils was 6.80 × 10^9^/L. There was no obvious abnormality in the absolute value and proportion of other blood cells. Among inflammation indicators, C-reactive protein (CRP) was 243.00 mg/L, and procalcitonin (PCT) was normal. Pathogenic microorganism examinations, including virus (including EB, cytomegalovirus (CMV), rubella, and herpes simplex viruses), toxoplasma, fungus, *tuberculosis*, and blood cultures, were all normal. Immunoglobulin IgG, IgA, IgM, and IgE were normal, complement C3, C4, rheumatoid factor, antineutrophil cytoplasmic antibody ANCA, antinuclear antibody Ana, anti-double-stranded DNA antibody, anti-SM antibody, anti-SSA, SSB antibody, anti-Ro52 antibody, anti-ScL-70 antibody, and anti-Jo-1 antibody were also normal. Lupus anticoagulant screening time (La 145.30 s), diagnosis time (La 232.30 s) and La1/La2 (at 1.40) were normal. immunoglobulin G4 subtype (IgG4) was normal, and there was no abnormality in urination and stool tests. The results of colour Doppler and transesophageal echocardiography were also normal, eliminating infective endocarditis. Chest computed tomography (CT) revealed minor inflammation in the right lower lung. Based on the above clinical manifestations, systematic examination results, and consultation among the Dermatology and Rheumatology Immunology Departments, the potential aetiology of recurrent fever and pleomorphic skin lesions was not identified.

To further determine the cause of these irregular phenomena in this patient, after obtaining the patient’s consent, left inguinal lymph node resection and biopsy were performed. The pathological diagnosis was lymphoid tissue hyperplasia with necrosis and neutrophil infiltration. Immunohistochemistry (IHC) found positivity for some markers, including CD20 (+, part), CD79a (+, minority), CD3 (+, part), CD5 (+, part), CD30 (+, minority), necrotic MPO (+, part), CD123 (+, part), and Ki-67 (+, 20–30%). Intriguingly, some positive bacilli were observed by acid-fast, silver hexamine, and periodic acid-Schiff (PAS) staining. However, DNA fragments of *Mycobacterium tuberculosis* (MTB) were not identified by qPCR. Flow cytometry showed no abnormally expressed populations of T, B, and NK lymphocytes. Combined with the results of pathological morphology, immunohistochemistry, and special staining, mycobacterial infection was considered, though it was difficult to determine which species.

**Fig. 2:**
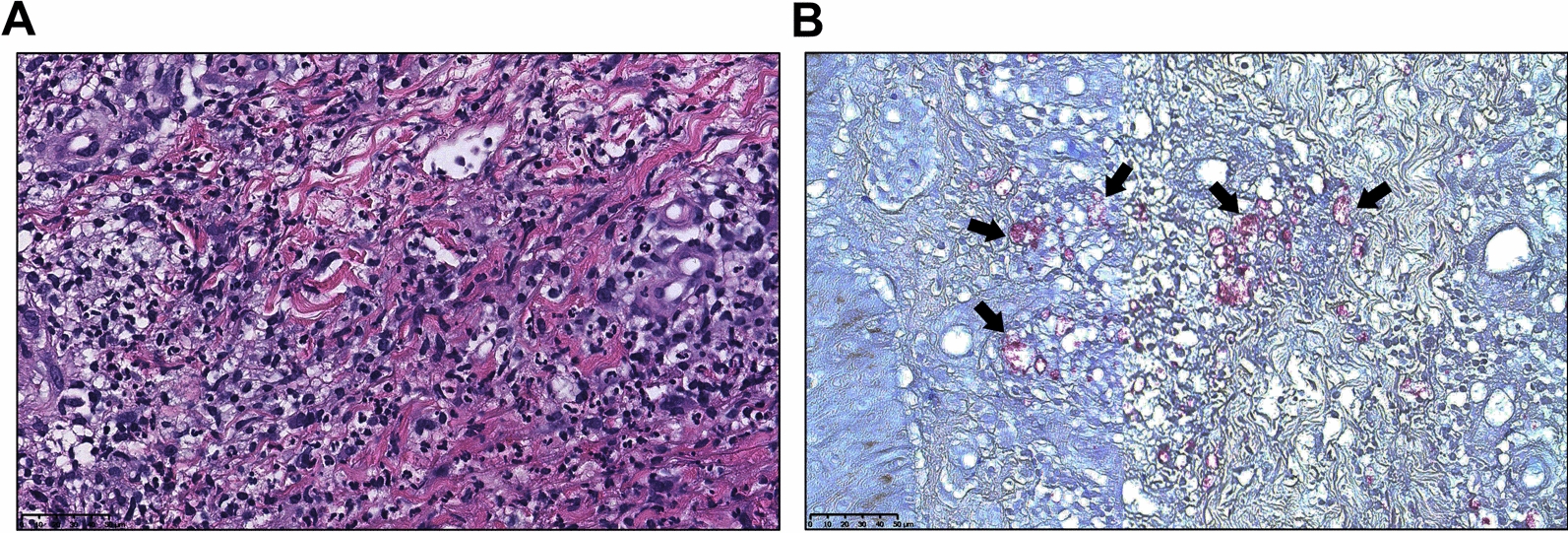
Pathological HE staining (**A**) and acid-fast staining (Ziehl–Neelsen) (**B**) of skin biopsy of the right leg. Scale bar: 20 μm

Considering the advantage of second-generation gene sequencing in distinguishing rare infectious diseases, a skin biopsy of the right leg flexor erythema was performed, the pathological examination and second-generation gene sequencing of fresh biopsy tissue were performed simultaneously. The pathological diagnosis showed a large number of lymphocytes, plasma cells, neutrophils, and histiocytes in the dermis and subcutaneous fat and around blood vessels. IHC also revealed positivity for some proteins, including CD3 (partial +), CD7 (partial +), CD8 (partial +), CD4 (partial +), and CD20 (partial +). Acid-fast staining showed that a large number of positive bacilli distributed in the dermis and subcutaneous fat (Fig. 2). However, the PCR did not detect any DNA fragments of *Mycobacterium tuberculosis*. In conclusion, our results were consistent with those of *Mycobacterium* infection; however, the species could not be confirmed by PCR or special antibody tests in our institute. The results of second-generation gene sequencing suggested the presence of *Mycobacterium leprae* (Table 1) but no other types of mycobacteria; thus, the patient was finally diagnosed as leprosy with erythema nodosum leprosum (ENL) [11, 12], characterized by erythema multiforme-like skin lesions, lymphadenopathy, fever, elevated CRP concentration, and histological perivascular infiltrate of neutrophils.

**Table 1.** High throughput gene detection results of pathogenic microorganisms

Type	Genus		Species	
	Latin name	Number of detected sequences	Latin name	Number of detected sequences
G^+^	*Mycobacterium*	3,79,485	*Mycobacterium leprae*	3,21,360

G + gram-positive bacteria

Since the pathogens of leprosy tend to affect the nerve system and loss of sensation in skin lesions is a cardinal sign in diagnosing leprosy, a careful nervous system physical examination was performed again, such as Shallow sensation of limbs, body and all skin lesions, which was evaluated in pain, touch and temperature. shallow reflex including corneal reflex, pharyngeal reflex and abdominal wall reflex; deep reflex including biceps brachii reflex, triceps brachii reflex, radial membrane reflex, knee reflex and heel reflex; pathological reflex including Hoffman sign, BABINSKY sign and Kirschner’s sign. Finally, the vision was also assessed. However, no abnormality was found.

The administration of prednisone acetate was stopped for this patient once the leprosy diagnosis was made. Due to a lack of leprosy treatment drugs in our hospital, this patient was referred to the Institute of Dermatology and Venereal Diseases. A therapy consisting of dapsone, rifampicin, and clofazimine was administered. The patient’s fever was controlled at 3 days after treatment, and her systemic erythema and superficial lymph node swelling were reduced at 3 months after treatment. At 1 year after treatment, the patient was followed up by telephone. Her temperature was normal, a few erythemas were present on the face, and her axillary lymph nodes were slightly larger, with no other uncomfortable symptoms.

## Discussion and conclusions

The patient’s skin lesions persisted for a long time. The primary clinical manifestation was systemic pleomorphic erythema, and there was no obvious abnormality in the peripheral nerves. Leprosy involves a variety of skin lesions, including spots or plaques. Few patients have pleomorphic or necrotic erythema. In severe cases, blisters or shallow ulcers occur in skin lesions. In the early stages, the superficial sensation of the skin lesions is normal or decreased, and the eyebrows were complete or not. In the late stages, the superficial sensation of the skin lesions may be disappeared, and the eyebrows may be shed [13]. Pleomorphic erythema is an acute inflammatory skin disease with target or iris skin lesions. Its clinical characteristics are an acute onset, a self-limited disease course, pleomorphic lesions, and easy recurrence. Pleomorphic erythema is common in young women [14]. The aetiology of erythema multiforme is complex. At present, it is generally considered an antigen–antibody reactive lesion, a type III hypersensitivity. Infection, drugs, and physical factors are common causes. Infections and drugs are the main pathogenic factors. Viral infection is the most common infectious factor, and streptococcal infection can also cause this disease [15]. Therefore, we should fully understand the clinical characteristics of leprosy, master its diagnostic points and methods, and then make a diagnosis of leprosy by carefully, comprehensively, and objectively analysing case characteristics in combination with the epidemic history, laboratory examination results, and clinical characteristics of diseases that need to be differentiated. In general, infectious, dermatology, neurology, rheumatology, and immunology teams are needed to assist in diagnosis and differential diagnosis [16].

Leprosy causes complex immune-mediated processes and clinical manifestations; it has had a low incidence rate in recent years and is prone to misdiagnosis. The misdiagnosis between leprosy and other non-leprosy diseases seriously delays accurate treatment, resulting in disability or even death [17, 18]. The probable reasons for misdiagnosis of leprosy are as follows. First, there are no special clinical manifestations or disease markers of leprosy, resulting in confusion with other skin lesion-related diseases. Second, the medical staff in basic hospitals lack a comprehensive and clear understanding of leprosy. In recent years, the incidence of leprosy has decreased, and as many younger doctors had never treated leprosy patients, they lack adequate vigilance when encountering leprosy. Finally, the lack of medical equipment and experienced pathologists in basic hospitals limits further investigation of aetiology. In the current case, erythema multiforme was not distinctive. The patient had no peripheral nerve damage. Skin lesions in leprosy are easily confused with skin diseases and autoimmune diseases. In clinical practice, misdiagnosis of leprosy is not uncommon. Avoiding leprosy misdiagnosis requires medical workers to have more understanding and vigilance, especially with sporadic cases [19].

The diagnosis of this patient was based on the two main clinical characteristics of superficial lymphadenopathy and systemic pleomorphic erythema. The multiple parts of lymph nodes and skin biopsy and subsequent routine pathological examination, immunohistochemistry, acid-fast, silver hexamine, and PAS staining provided key information for final diagnosis. Overall, positive histopathological results are valuable for diagnosing leprosy. However, histopathological examination takes a long time, and many factors, such as the sampling site, section, and assessment, will affect the judgement of results. More importantly, current pathology limitations render diagnosing the species of *Mycobacterium* difficult. Considering the positive acid-fast staining, an expert consensus has suggested that second-generation sequencing can significantly increase the detection rate of pathogens for rare and complex infectious diseases, and sporadic cases sometimes lack exposure and travel history. Therefore, second-generation gene sequencing has become an important means of detection. In the present case, second-generation gene sequencing was used simultaneously to improve the positive rate and accuracy of the diagnosis. This case also shows that second-generation sequencing can assist in diagnosing complicated clinical infections and that traditional laboratory examination and second-generation gene sequencing can verify each other to obtain faster and more accurate test results [19]. However, second-generation gene sequencing cannot replace traditional detection methods but should be used as an auxiliary inspection method when searching for infectious pathogens. The diagnosis of this case fully reflects that the combination of a single examination can improve the timeliness and accuracy of diagnosis.

## Data Availability

The datasets used and/or analysed during the current study are available from the corresponding author on reasonable request.

## Abbreviations

PAS: Periodic acid-Schiff
WHO: World Health Organization
CRP: C-reactive protein
PCT: Procalcitonin
IHC: Immunohistochemistry
